# Editorial: Sustainability of physical activity interventions and public health

**DOI:** 10.3389/fpubh.2022.1115411

**Published:** 2022-12-21

**Authors:** Erica Y. Lau, Maureen C. Ashe, Sharon E. Taverno Ross, Russell R. Pate

**Affiliations:** ^1^Department of Emergency Medicine, University of British Columbia, Vancouver, BC, Canada; ^2^Department of Family Practice, University of British Columbia, Vancouver, BC, Canada; ^3^Department of Health and Human Development, University of Pittsburgh, Pittsburgh, PA, United States; ^4^Department of Exercise Science, University of South Carolina, Columbia, SC, United States

**Keywords:** physical activity, implementation science, health promotion (HP), sustainability, maintenance

Physical activity is today's “best buy” in public health (1). Participation in regular physical activity could prevent and slow the progression of a wide range of non-communicable diseases, such as heart disease, stroke, diabetes and breast and colon cancer (2, 3). To obtain these health benefits, it is recommended that adults (aged 18–64 years) participate in at least 150 min/week of moderate-to-vigorous physical activity; children and adolescents aged 6 through 17 years should accumulate 60 min or more of moderate-to-vigorous physical activity daily (2, 4). Yet, 27.5% of adults and 81% of adolescents fail to meet recommended physical activity guidelines. That is, they are physically inactive (5, 6).

Physical inactivity is responsible for major health and economic burden. Globally, physical inactivity is attributed to up to 8% of deaths and non-communicable diseases and an international $ (INT$) 53.8 billion health-care systems cost (7, 8). The World Health Organization published the Global Action Plan on Physical Activity with the goal of a 15% relative reduction in the global prevalence of physical inactivity in adults and adolescents by 2030.

Action toward addressing physical inactivity is possible because there is evidence of what works. The Toronto Charter for Physical Activity outlines 7 best investments that work for physical activity, such as transport policy and systems community-wide programs (9). Over the years, evidence on effective physical activity interventions for different settings and populations has been growing. However, there has been a failure to sustain effective interventions at the population level (10). Sustainability broadly refers to continued use of program components at sufficient intensity to achieve the desired program goals and population outcomes (11). Failure to invest in sustainable interventions squanders start-up investments and prevents the realization of program benefits (12).

In the past several years, the Coronavirus (COVID-19) SARS-CoV-2 disrupted our daily routines. To contain and reduce the spread of the virus, national governments across the globe introduced various public health measures, such as social distancing and quarantine, that substantially limited opportunities for physical activity (13, 14). We witnessed the detrimental effects of physical inactivity on physical and mental health, reinforcing the urgent need to promote physical activity for population health (15). Identifying sustainable physical activity interventions and learning from their experiences has never been so important.

The aim of this Special Research Topic was to provide an opportunity to report on the latest findings for how to sustain implementation and the health impact of effective physical activity interventions. It consists of four publications, including one protocol study and three original research studies. Below, we provide a short summary for each publication.

The protocol study from Till et al. aims to use theory and frameworks to sustain two previously tested physical activity interventions in 15 new communities focusing on older adults at risk for dementia and women with difficult life situations, such as living with unemployment and/or low income. The authors will use a participatory approach to support community partners in delivering the intervention. They will evaluate outcomes guided by RE-AIM (16) and the Consolidated Framework for Implementation Research (CFIR) (17). This study provides an implementation evaluation framework, and a list of outcomes and tools for other studies to reference, adopt, test, and modify.

In the large longitudinal study by Chrismas et al., the researchers analyzed step counts from a pedometer or smartphone app from >16,000 people from Qatar during a 7-year community-based program. The two main outcomes were absolute adherence to the intervention (as a percentage of days with data over time), and retention of the intervention (length of time a participant stayed in the program, e.g., days/7 years). The authors discussed the challenges and potential strategies for sustaining usage in large-scale wearable technology-based physical activity interventions.

In the outcome evaluation of the Active Hertz program from Chater et al., the researchers shared the findings from a large, UK-based, multi-phased, longitudinal 12-month physical activity program for 717 adults at risk of cardiovascular disease. The study found that this community-based intervention with frequent behavior change training and supervision can help adults at risk of cardiovascular disease sit less and move more. The authors highlighted that funding and building capacity for physical activity in health systems (e.g., physical activity specialist) would be keys to sustaining the program at scale in “real-world” settings.

Finally, Lau et al. examined the sustained implementation of a commercial mHealth app in two Canadian provinces. In this retrospective observational study, researchers investigated real-world app usage from >41,000 adults over 12 months. This study provided empirical evidence that long-term mHealth app usage is possible and underscored the importance of considering intermittent usage patterns when designing and evaluating sustainable mhealth interventions.

Taken together, the research articles in this Special Research Topic provide some theory and frameworks to guide interventions, had large study sample sizes, ranging from 717 to >41,000 participants, and considered several equity-related factors. Findings offer insights into key factors to consider when designing and sustaining physical activity interventions; and studies discussed the challenges with intervention sustainment in “real-world” settings.

## Author contributions

MA and EL drafted the editorial. SR and RP provided critical feedback on the content and organization of the editorial. All authors read and approved the submitted editorial and have agreed to be personally accountable for their contribution.
